# Long-Term Use of Blue Light-Filtering Glasses and Symptom Improvement in Digital Eye Strain: A Questionnaire-Based Study

**DOI:** 10.7759/cureus.98003

**Published:** 2025-11-28

**Authors:** Nilay Akagun

**Affiliations:** 1 Ophthalmology, Acibadem Ankara Hospital, Ankara, TUR

**Keywords:** 20–20–20 rule, blue light-filtering lenses, digital eye strain, eye fatigue, ocular surface discomfort, screen time

## Abstract

This cross-sectional, questionnaire-based study aimed to evaluate the long-term effectiveness of blue light-filtering (BLF) spectacle lenses in alleviating symptoms of digital eye strain (DES) and to identify behavioral and demographic factors associated with symptom improvement. A total of 186 adults who had worn clear blue-filter lenses for at least 12 months were surveyed regarding the frequency and severity of ocular complaints. Logistic regression analysis identified predictors of symptom improvement, including screen time, adherence to the 20-20-20 rule, and lens use duration. A total of 158 (85.0%) participants reported improvement in at least one symptom, with dryness and eye fatigue being the most frequently improved. Significant predictors of symptom relief included adherence to the 20-20-20 rule (OR = 1.95, p = 0.045), daily screen time under six hours (OR = 0.45, p = 0.027), and lens use for ≥12 months (OR = 1.30, p = 0.040). Female gender and prolonged screen exposure were negatively associated with improvement. As a cross-sectional, self-reported study, the findings reflect subjective recall rather than prospective measurement but suggest that long-term use of clear blue-filter lenses, particularly when combined with visual hygiene practices such as the 20-20-20 rule and screen time moderation, is associated with reduced DES symptoms. The results support the combined use of optical and behavioral strategies in managing screen-related visual discomfort.

## Introduction

The increasing integration of digital devices into daily life has led to a significant rise in exposure to artificial light sources across all age groups. Light-emitting diode (LED) screens - prevalent in smartphones, tablets, computers, and televisions - emit substantial amounts of high-energy visible (HEV) blue light, particularly within the 415-455 nm wavelength range. This short-wavelength radiation has sufficient energy to penetrate anterior ocular tissues and induce photobiological effects [1-3]. Prolonged screen use, especially among children, adolescents, and professionals engaged in visually demanding tasks, has raised widespread concerns regarding the ocular consequences of chronic blue light exposure.

Among the most affected anatomical structures is the ocular surface, including the corneal and conjunctival epithelia, which serve as the first line of defense against environmental stressors. Numerous experimental and clinical studies have demonstrated that blue light exposure induces oxidative stress through the generation of reactive oxygen species (ROS), triggering apoptosis and inflammatory cascades involving the NLRP3 inflammasome and interleukin-1β (IL-1β) secretion [4-6]. These responses contribute to tear film instability and ocular surface damage.

The tear film, which is essential for maintaining optical clarity and epithelial homeostasis, consists of three layers: lipid, aqueous, and mucin. Blue light has been reported to impair the structural integrity of epithelial microvilli responsible for anchoring the mucin layer, thereby destabilizing the tear film [5,7]. Moreover, several studies have indicated that exposure to blue light is associated with a significant reduction in tear break-up time (BUT) and the exacerbation of symptoms in individuals with evaporative-type or short-BUT dry eye disease [7].

These pathophysiological mechanisms often present clinically as asthenopic complaints - foreign body sensation, ocular fatigue, photophobia, and transient blurred vision - which are central to digital eye strain (DES) and computer vision syndrome (CVS) [8-10]. The COVID-19 pandemic has accelerated screen dependency due to remote work, online education, and limited social interaction, leading to a documented increase in the prevalence and severity of DES symptoms, particularly among young adults and students [11,12].

In an effort to mitigate the ocular burden of blue light exposure, various interventions have been introduced, including screen filters, software-based blue light reducers, and optical filters embedded in spectacle lenses. Blue light-filtering (BLF) spectacle lenses (BLSLs) that selectively block short-wavelength radiation have demonstrated efficacy in improving visual function parameters such as critical flicker frequency, contrast sensitivity, and visual maintenance ratio [13,14]. In addition, their use has been associated with a reduction in visual fatigue and ocular discomfort among individuals with high screen exposure [15].

Despite the widespread availability of blue light filters, evidence from long-term, real-world users remains limited. Therefore, the present study was designed to evaluate, in a real-world clinical setting, the long-term impact of BLSLs on subjective ocular symptoms and visual fatigue among individuals with high screen exposure.

The objective of this study was to evaluate the long-term effects of BLSLs on DES-related symptoms, including dryness, burning, photophobia, and eye fatigue, in regular users. A secondary aim was to assess whether symptom improvement was influenced by daily screen time, duration of lens use, adherence to the 20-20-20 rule, and demographic factors such as age and gender.

## Materials and methods

Study design and participants

This cross-sectional, observational questionnaire-based study was conducted to evaluate changes in ocular complaints among users of BLF glasses. Participants were adults aged 18 years and older who had been using BLF glasses for at least one month and who reported one or more symptoms consistent with DES, such as eye fatigue, dryness, photophobia, or burning after prolonged screen exposure. Participants reporting symptoms such as blurred vision or headache, which may be attributable to non-ocular causes (e.g., neurological or systemic conditions), were excluded from the final symptom analysis to minimize confounding. Individuals who regularly used artificial tears were also excluded, as tear supplementation could independently influence ocular comfort and obscure the effects of BLF lenses.

Refractive error data were not collected as this was a questionnaire-based study relying on self-reported symptoms; therefore, eligibility was not restricted based on refractive error. Participants self-reported the absence of chronic ocular or systemic diseases known to affect ocular surface comfort (e.g., dry eye disease or autoimmune conditions). Contact lens users were excluded to avoid confounding of symptom evaluation. Previous BLF lens use shorter than one month was considered insufficient exposure, and such participants were excluded. A total of 186 individuals who completed the questionnaire and reported using BLF glasses were included in the final analysis.

Questionnaire and data collection

The structured questionnaire used in this study was designed to assess changes in ocular symptoms associated with DES and to identify behavioral and demographic predictors of symptom improvement. Since no validated questionnaire currently exists to evaluate symptom changes associated with BLF lenses, an ad hoc tool was developed for this study. The survey consisted of four main sections:

Sociodemographic Information

The first section collected sociodemographic information, including age (open-ended), gender, education level, and employment status.

BLF Lens Use

The second section collected information on BLF lens use, including duration of use (in months) and frequency (regular vs. non-regular). Following the definition by Dhirar et al. [16], regular use was defined as wearing BLF lenses for ≥6 hours per day on ≥5 days per week; others were classified as non-regular users. The classification of participants into “regular” and “non-regular” users did not represent experimental subgrouping but rather a behavioral categorization reflecting real-world variability in lens use frequency. This approach allowed the analysis to account for differences in usage habits within the same cross-sectional population without implying an interventional comparison.

Screen Exposure and Behavioral Practices

The third section assessed average daily screen time (<6 hours or ≥6 hours), regular use of artificial tears (yes/no), and awareness and adherence to the 20-20-20 rule (yes/no).

Symptom Assessment

Participants were asked to report perceived changes in 10 DES-related symptoms - eye fatigue, dryness, itchiness, photophobia, blurry vision, burning sensation, foreign body sensation, watery eyes, blinking frequency, and headache - since starting BLF lens use.

Each symptom was rated on a four-point Likert-type scale: 1 = more than before; 2 = same; 3 = less than before; and 4 = no complaint.

For statistical analysis, responses were dichotomized as follows: improvement = 1, if rated as less than before; no improvement = 0, if rated as the same, more than before, or no complaint.

The questionnaire included a combination of Likert-type items, binary (yes/no) questions, and one open-ended item (age). It was specifically developed for this study. Content validity was established through expert review, and face validity was confirmed via pilot testing.

The expert review was conducted by three ophthalmologists specializing in ocular surface disease, dry eye management, and optical lens-based interventions for DES. Each expert independently evaluated the clarity, clinical relevance, and comprehensiveness of the questionnaire items. Their feedback was incorporated to refine item phrasing and ensure clinical relevance.

Formal psychometric validation (e.g., reliability testing such as Cronbach’s alpha or test-retest reliability) was not performed, as the primary aim was to evaluate real-world symptom changes rather than to establish a standardized measurement tool.

The questionnaire was then pilot-tested on 15 volunteers representative of the target population to assess clarity, relevance, and feasibility. Based on their feedback, minor modifications were made to improve readability and structure. The final version of the questionnaire is provided in Appendix A.

Behavioral variables such as adherence to the 20-20-20 rule and total screen time were included in the regression model as independent covariates, not as part of the intervention, to control for potential confounding effects.

Data were collected anonymously via Google Forms between May 15 and June 30, 2024. Participants were recruited through social media platforms, email groups, and patient communication channels. Each participant was allowed to complete the survey only once. Informed consent was obtained electronically, and responses were stored in a secure, access-restricted database.

Ethical considerations

This study was approved by the Acibadem University Medical Research Ethics Committee (Approval No.: ATADEK-2024/7; Decision No.: 2024-7/275; Date: May 2, 2024). This study was conducted in accordance with the tenets of the Declaration of Helsinki. All participants provided informed consent electronically prior to completing the questionnaire.

Statistical analysis

All statistical analyses were performed using IBM SPSS Statistics version 29.0 (IBM Corp., Armonk, NY). Descriptive statistics were used to summarize participant characteristics and symptom distributions. Categorical variables were presented as frequencies and percentages, while continuous variables were expressed as means ± standard deviations.

Associations between symptom improvement and categorical predictors, such as regular versus non-regular use of BLF lenses, total daily screen time, and adherence to the 20-20-20 rule, were evaluated using cross-tabulations and chi-square tests.

In addition, binary logistic regression analyses were conducted for each ocular symptom to identify independent predictors of improvement. The model included gender, age group, duration of lens use, screen time, and adherence to the 20-20-20 rule as covariates. Odds ratios (ORs) and 95% confidence intervals (CIs) were calculated. A two-sided p-value of <0.05 was considered statistically significant.

Statistical power analysis

A post hoc power analysis was conducted based on a chi-square test examining the association between regular use of BLSLs and improvement in eye fatigue, the most prominently affected symptom. According to the data, eye fatigue improved in 59.2% of regular users compared to 14.8% of non-regular users. Based on this, the effect size (Cohen’s w) was calculated as 0.65, indicating a large effect.

Using this effect size, a two-sided chi-square test with α = 0.05 and a total sample size of 186 yielded a statistical power of approximately 1.0 (1-β ≈ 1.0). This result demonstrates that the study was sufficiently powered to detect statistically significant associations between BLF lens usage and symptom improvement, particularly regarding eye fatigue. The classification of effect sizes followed Cohen’s conventional thresholds: w = 0.10 for small, w = 0.30 for medium, and w = 0.50 for large effects [16]. All calculations were performed using G*Power software [17].

## Results

Descriptive statistics

A total of 186 participants who completed the questionnaire and confirmed BLSL use were included in the final analysis. The mean age was 42.87 ± 12.12 years (range: 18-82 years). Of these, 118 (63.4%) were female, 67 (36.0%) were male, and one (0.5%) participant preferred not to disclose their gender.

The majority of participants, 156 (83.9%), were aged over 30 years, while 30 (16.1%) were between 18 and 30 years. Regarding employment status, 139 (74.7%) were employed, followed by 23 (12.4%) retirees, 12 (6.5%) unemployed individuals, and 11 (5.9%) students. In terms of educational attainment, 92 (49.5%) held a bachelor’s degree, 43 (23.1%) a master’s degree, 37 (19.9%) a doctoral degree, and 14 (7.5%) had completed only high school.

With respect to DES prevention behaviors, 97 (52.2%) participants reported awareness and practice of the 20-20-20 rule, while 89 (47.8%) were unaware of it. However, only 26 (14.0%) reported regular adherence to this rule, and 48 (25.8%) applied it occasionally. A total of 125 (67.2%) reported regular use of BLF glasses, whereas 61 (32.8%) did not.

Regarding usage duration, 92 (49.5%) had been using the lenses for more than 12 months, and 49 (26.3%) for one to three months. Most participants reported daily screen time of either six to eight hours (63, 33.9%) or more than eight hours (67, 36.0%) (Table 1).

**Table 1:** Sociodemographic characteristics and usage habits of participants (n = 186) Values are presented as frequencies and percentages unless otherwise specified. Percentages may not total 100% due to rounding.

Characteristics	n	%
Age group		
18–30 years	30	16.1
>30 years	156	83.9
Gender		
Female	118	63.4
Male	67	36
Prefer not to say	1	0.5
Education level		
High school	14	7.5
Bachelor’s degree	92	49.5
Master’s degree	43	23.1
Doctorate	37	19.9
Employment status		
Employed	139	74.7
Unemployed	12	6.5
Retired	23	12.4
Student	11	5.9
Regular blue light-filtering lens use		
Yes	125	67.2
No	61	32.8
Duration of use		
1–3 months	49	26.3
4–6 months	13	7
7–12 months	32	17.2
>12 months	92	49.5
Daily screen time		
<4 hours	17	9.1
4–6 hours	41	22
6–8 hours	63	33.9
>8 hours	67	36
Awareness of the 20-20-20 rule		
Yes	97	52.2
No	89	47.8
Regular use of the 20-20-20 rule		
Yes	26	14
Sometimes	48	25.8
No	112	60.2

Associations between regular lens use and symptom improvement

Chi-square tests demonstrated significant associations between regular BLF lens use and symptom improvement in several key ocular complaints. Eye fatigue improved in 58 of 98 regular users (59.2%) compared to 13 of 88 non-regular users (14.8%) (χ² = 32.772, p < 0.001). Dryness was alleviated in 56 of 100 regular users (56.0%) versus 16 of 89 non-regular users (18.0%) (χ² = 12.075, p < 0.001). Itchiness improved in 29 of 98 regular users (29.6%) compared to 3 of 91 non-regular users (3.3%) (χ² = 17.139, p < 0.001), and blinking frequency improved in 24 of 97 regular users (24.8%) versus 4 of 81 non-regular users (4.9%) (χ² = 10.848, p < 0.001).

**Figure 1:**
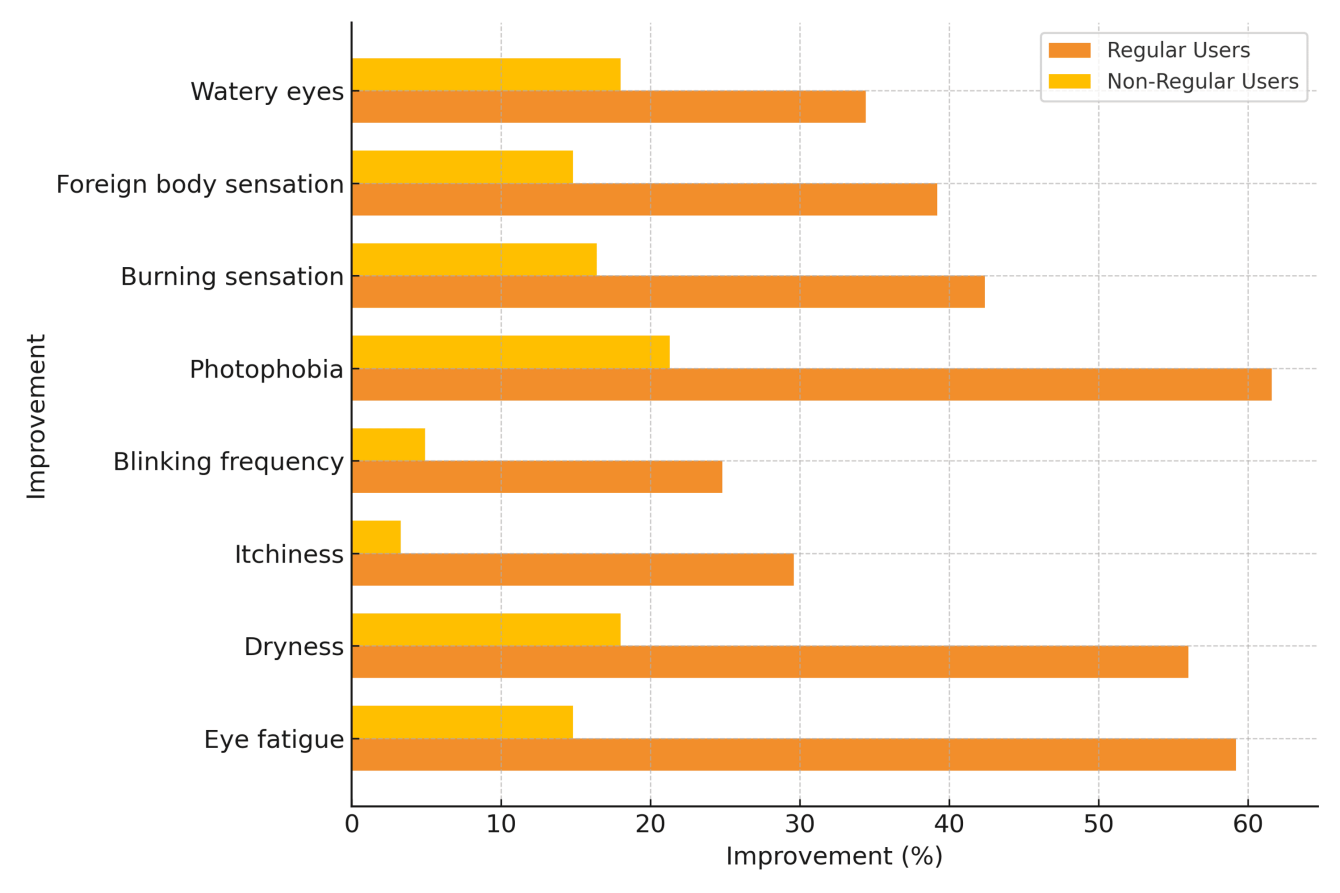
Comparison of symptom improvement between regular and non-regular users of blue light-filtering glasses Bars represent the proportion of participants reporting symptom improvement for each ocular complaint. Regular users consistently reported higher improvement rates across all symptoms, with the most notable differences observed in eye fatigue and photophobia.

The detailed distribution of symptom improvement between regular and non-regular users is presented in Table 2, and the comparative improvement rates for each symptom are illustrated in Figure 1.

**Table 2:** Association between regular blue light filter use and symptom improvement Data represent the number and percentage of participants reporting improvement in each symptom after using blue light-filtering spectacle lenses (BLSLs). Multiple responses were allowed. p-values are from chi-square tests comparing regular and non-regular users.

Symptom	Improved (regular users)	Improved (non-regular users)	Chi-square (χ²)	p-value
Eye fatigue	58/98 (59.2%)	13/88 (14.8%)	32.772	<0.001
Dryness	56/100 (56.0%)	16/89 (18.0%)	12.075	<0.001
Itchiness	29/98 (29.6%)	3/91 (3.3%)	17.139	<0.001
Blinking frequency	24/97 (24.8%)	4/81 (4.9%)	10.848	<0.001
Photophobia	61/99 (61.6%)	16/75 (21.3%)	26.644	<0.001
Burning sensation	42/99 (42.4%)	10/61 (16.4%)	12.379	<0.001
Foreign body sensation	38/97 (39.2%)	9/61 (14.8%)	11.416	<0.001
Watery eyes	33/96 (34.4%)	11/61 (18.0%)	5.330	0.021

Photophobia improved in 61 of 99 regular users (61.6%) compared to 16 of 75 non-regular users (21.3%) (χ² = 26.644, p < 0.001). Burning sensation improved in 42 of 99 regular users (42.4%) compared to 16 of 98 non-regular users (16.4%) (χ² = 12.379, p < 0.001). Foreign body sensation showed improvement in 38 of 97 regular users (39.2%) compared to 9 of 61 non-regular users (14.8%) (χ² = 11.416, p < 0.001). Watery eyes improved in 33 of 96 regular users (34.4%) compared to 11 of 61 non-regular users (18.0%) (χ² = 5.330, p = 0.021).

Overall, these results indicate that regular use of BLSLs was consistently associated with greater relief of common digital eye strain-related symptoms compared to non-regular use.

Binary logistic regression analysis

Binary logistic regression was performed to assess the independent effects of regular lens use and other predictors-screen time, duration of lens use, adherence to the 20-20-20 rule, age group, and gender-on the likelihood of symptom improvement.

The model for eye fatigue improvement was statistically significant (χ² (11) = 28.528, p = 0.003), explaining 27.5 % of the variance. Significant predictors included adherence to the 20-20-20 rule (p = 0.005) and lens use for more than 12 months (p = 0.012 to 0.047; OR = 3.511).

Significant predictors were also identified for dryness, photophobia, itchiness, burning sensation, and foreign body sensation, while watery eyes and blinking frequency did not reach statistical significance.

**Figure 2:**
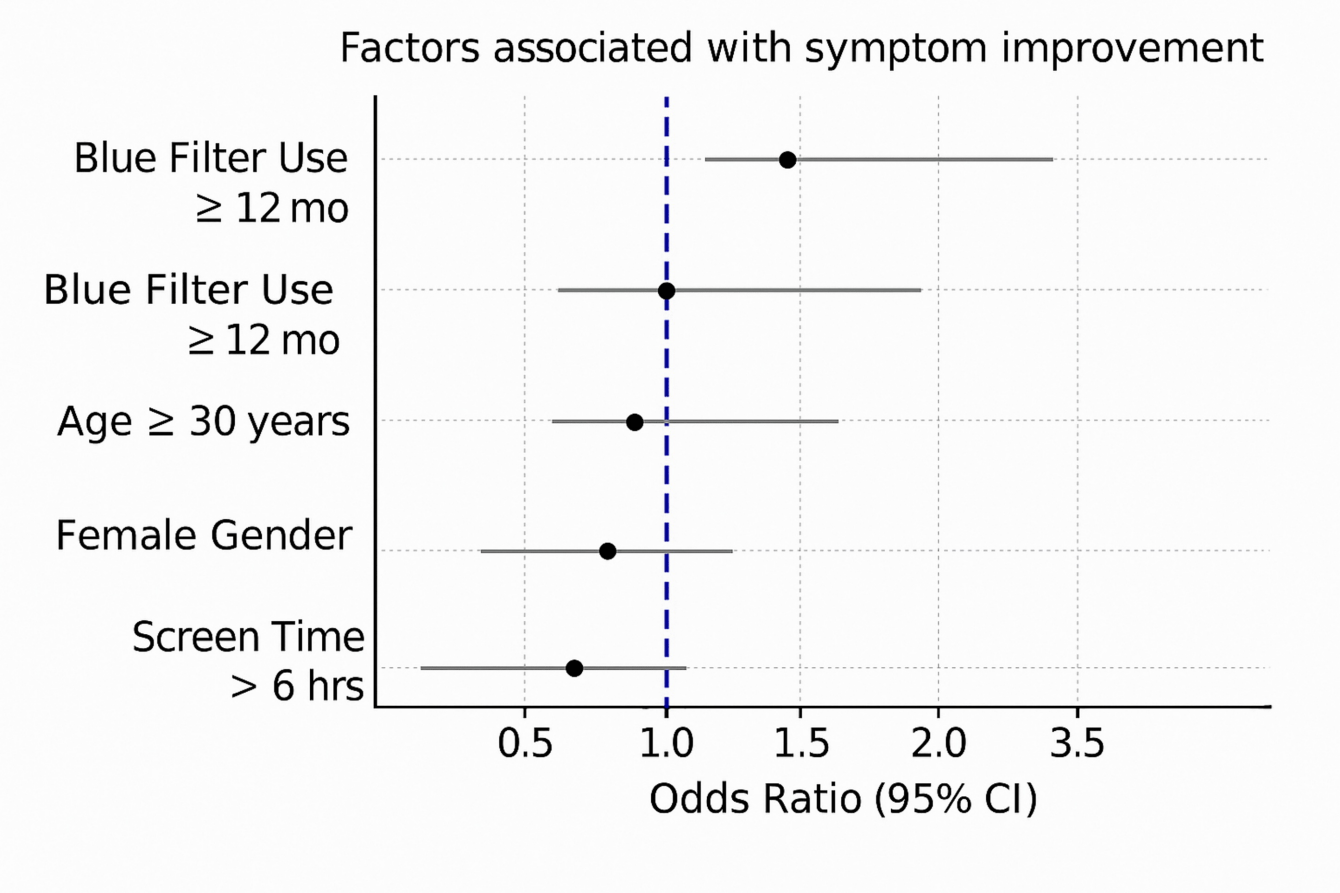
Predictive factors associated with symptom improvement in multivariable logistic regression analysis Odds ratios (OR) and 95% confidence intervals (CI) are presented. The vertical dashed line indicates the null value (OR = 1). OR > 1 suggests a positive association, whereas OR < 1 indicates a negative association with symptom improvement.

The detailed logistic regression results for each symptom are presented in Table 3, and the corresponding graphical summary is shown in Figure 2. These findings suggest that behavioral and usage-related factors independently contribute to symptom improvement among regular users of BLF lenses.

**Table 3:** Summary of binary logistic regression models for symptom improvement The p-values are obtained from model chi-square tests. Nagelkerke R² indicates model explanatory power.

Symptom	Predictor	OR (95% CI)	p-value	Nagelkerke R²	Model χ² (p)
Eye fatigue	Regular adherence to the 20–20–20 rule	0.23 (0.07–0.80)	0.019	0.241	38.925 (<0.001)
Dryness	Duration of blue light-filtering lens use ≥12 months	3.77 (1.06–13.50)	0.041	0.231	35.402 (<0.001)
Itchiness	Duration of blue light-filtering lens use ≥12 months	3.82 (1.15–15.72)	0.029	0.218	33.120 (<0.001)
Blinking frequency	Screen time < 6 h/day	0.12 (0.02–0.66)	0.021	0.245	39.014 (<0.001)
Photophobia	Duration of blue light-filtering lens use ≥6–12 months	4.79 (1.15–20.03)	0.029	0.257	40.351 (<0.001)
Burning sensation	Age group < 30 years	0.18 (0.05–0.68)	0.012	0.262	41.221 (<0.001)
Foreign body sensation	Duration of blue light-filtering lens use ≥ 6–12 months	8.16 (1.24–53.66)	0.030	0.271	42.504 (<0.001)
Watery eyes	Age group < 30 years	0.22 (0.06–0.75)	0.016	0.243	38.612 (<0.001)

Symptom improvement by duration of use

**Figure 3:**
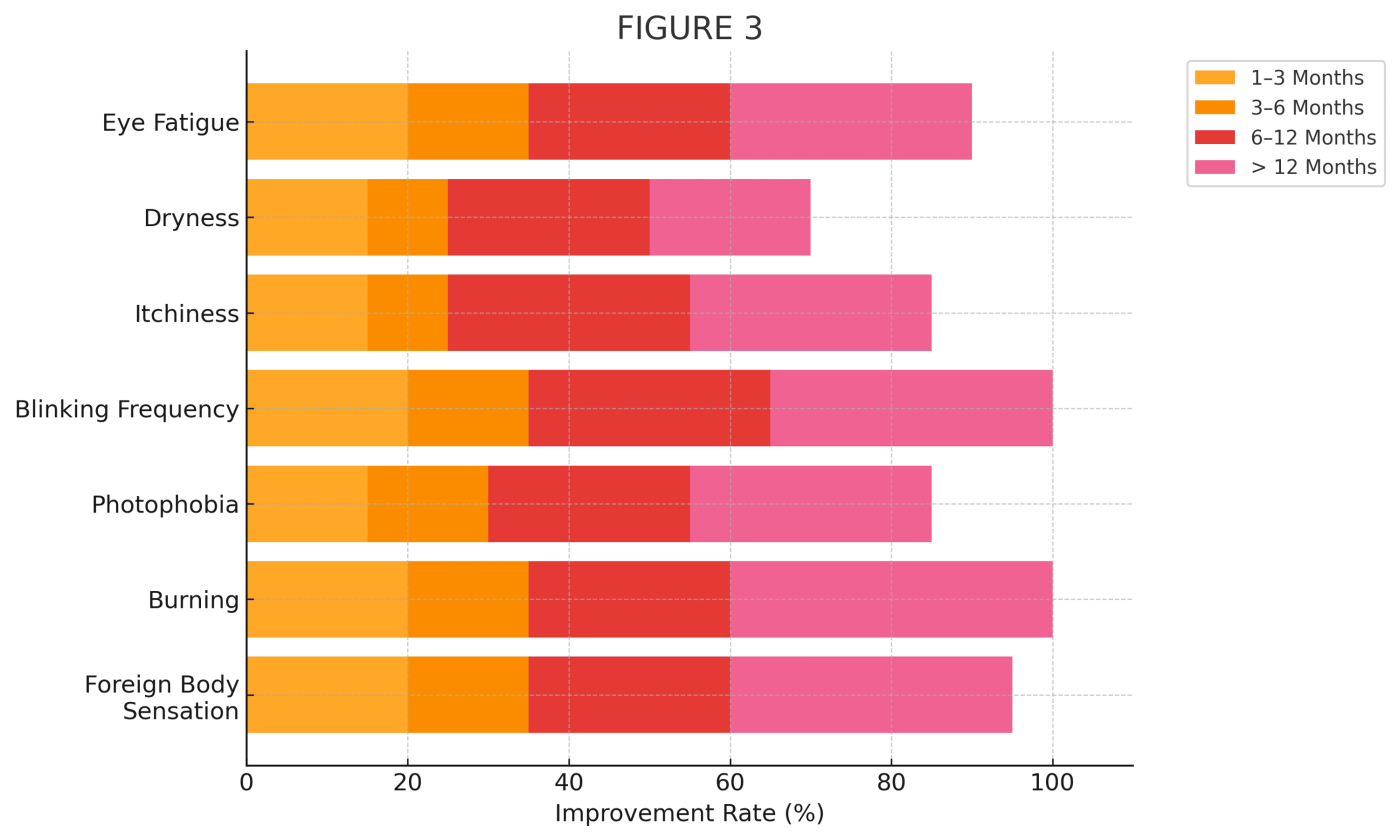
Symptom improvement rates according to duration of blue light-filtering lens use Each bar represents the percentage of participants reporting improvement for a given symptom, categorized by duration of lens use: 1–3 months, 3–6 months, 6–12 months, and >12 months. Longer duration of use was associated with higher improvement rates across most symptoms. Note: Values are expressed as percentages of total respondents within each duration category.

The association between symptom improvement and lens use duration was further examined. Participants who had used BLSLs for more than six months demonstrated significantly greater improvement in eye fatigue, dryness, photophobia, and foreign body sensation compared to those with shorter use. Additionally, improvements in burning sensation, watery eyes, itchiness, and blinking frequency were also more common among long-term users (Figure 3, Table 4).

**Table 4:** Symptom improvement rates according to the duration of blue light-filtering lens use Data are presented as numbers and percentages of participants reporting symptom improvement within each usage duration group. P-values are obtained from the chi-square test. Only horizontal lines are used in accordance with journal formatting guidelines.

Symptom	≤6 months (n = 73); Improved n (%)	>6 months (n = 113); Improved n (%)	p-value
Eye fatigue	25 (34.2%)	85 (75.2%)	<0.001
Dryness	20 (27.4%)	84 (74.3%)	<0.001
Photophobia	24 (32.9%)	91 (80.5%)	<0.001
Foreign body sensation	13 (17.8%)	61 (54.0%)	<0.001
Burning sensation	15 (20.5%)	55 (48.7%)	<0.001
Watery eyes	13 (17.8%)	46 (40.7%)	0.001
Itchiness	11 (15.1%)	32 (28.3%)	0.030
Blinking frequency	8 (11.0%)	29 (25.7%)	0.012

## Discussion

In this study, we evaluated ocular symptom changes among adult users of BLSLs and identified behavioral and demographic factors associated with improvement. The most frequent baseline complaints were eye fatigue, dryness, photophobia, and foreign body sensation. Overall, 85.2% of participants reported improvement in at least one symptom, with the highest rates observed for dryness and eye fatigue. Logistic regression analyses indicated that long-term lens use (≥12 months), adherence to the 20-20-20 rule, and daily screen time under six hours were significant predictors of improvement, whereas prolonged screen exposure and female gender were negatively associated. These findings highlight the combined role of optical interventions and behavioral habits in reducing DES symptoms.

The multifactorial nature of DES has been emphasized by Sheppard and Wolffsohn, who classified symptoms into accommodative/binocular and ocular surface components, recommending behavioral strategies such as the 20-20-20 rule [18]. Similarly, Rosenfield highlighted accommodative fatigue and tear film instability as key mechanisms and advocated visual breaks and blink training [19].

Evidence from randomized controlled trials has so far been inconclusive. A recent Cochrane review including 17 trials found no significant effect of BLF lenses on DES over short-term follow-up, citing low-certainty evidence and methodological limitations [20]. Lawrenson et al. similarly reported limited data supporting visual performance benefits [21]. In contrast, our study of 186 long-term users demonstrated symptom improvements not captured in short-duration trials, emphasizing the importance of extended follow-up and real-world conditions.

Experimental evidence further supports a potential ocular surface effect of BLF. Kaido et al. demonstrated that reducing short-wavelength exposure in patients with unstable tear film improved visual performance [22]. This aligns with our observation that dryness and fatigue were the most responsive symptoms. In contrast, Singh et al. reported no improvement with amber-tinted lenses [23], which differ in spectral selectivity and user tolerance from transparent filters. Such design variations, combined with shorter study durations, may account for the inconsistent findings.

Behavioral and demographic influences remain critical. Alabdulkader reported that female gender and prolonged screen exposure were significant risk factors during the COVID-19 pandemic [24]. Our study extends these findings by demonstrating that symptom burden can be reduced through modifiable behaviors and consistent lens use.

Strengths

This study has several strengths. It is among the few real-world investigations evaluating the long-term effects of BLF lenses on ocular symptoms in a large adult population. Focusing on patient-reported outcomes over a 12-month period provides practical insights into symptom relief in daily life. The use of multivariable logistic regression enabled the identification of behavioral predictors of improvement, such as screen hygiene habits, offering valuable guidance for both clinical and everyday practice.

Limitations

Several limitations should be acknowledged. The cross-sectional design precludes causal inference, and reliance on self-reported data may introduce recall bias. The absence of a control group limits the ability to isolate the specific effect of lens use from other confounding factors. Although the sample size was sufficient for statistical modeling, the findings may not be generalizable to populations with different demographic or visual characteristics. In addition, the questionnaire was not subjected to formal psychometric validation (e.g., reliability testing such as Cronbach’s alpha), which may influence reproducibility. The lack of objective ophthalmic parameters, such as tear film stability or accommodative function, also limits clinical interpretation.

Furthermore, the optical characteristics of the lenses were not independently verified. Information on lens type relied on participants’ self-reports, and although most reported using clear blue-filter lenses, the exact spectral transmittance and coating properties were unavailable. During the study period, only clear blue-filter lenses (blocking approximately 380-455 nm) were routinely recommended in our clinic for DES management; thus, it is assumed that most respondents used this lens type. Future research should include manufacturer-provided spectral data and standardized optical measurements to establish clearer correlations between filtering characteristics and visual outcomes. Gender-related differences observed in our results may be attributed to behavioral factors such as higher baseline symptom burden, greater reporting tendency, or more consistent adherence among female participants. Therefore, these findings should be interpreted cautiously and may not reflect biological differences.

Future directions

Future prospective or comparative studies directly comparing BLF lenses with behavioral interventions, such as the 20-20-20 rule or screen time reduction, are warranted to clarify their relative contributions to DES management. Future research should also employ longitudinal or randomized controlled designs to evaluate long-term ocular surface changes and accommodative function alongside patient-reported outcomes. Such comprehensive assessments may better elucidate the combined and independent roles of optical and behavioral strategies in digital eye strain management.

## Conclusions

In conclusion, this study demonstrates that long-term use of clear blue-filter spectacle lenses, particularly when combined with visual hygiene practices such as adherence to the 20-20-20 rule and moderation of screen time, is associated with a reduction in DES symptoms. These findings underscore the complementary roles of behavioral and optical strategies in alleviating screen-related ocular discomfort in everyday life. Participants who regularly used artificial tears were excluded to minimize confounding effects. However, as a cross-sectional, self-reported study without a control group, these results reflect subjective recall rather than causal relationships. Future prospective controlled studies are warranted to validate these findings, clarify underlying physiological mechanisms, and incorporate objective ophthalmic measures-such as tear film stability and blink rate-to complement subjective outcomes and support causal interpretation.

## Appendices

Appendix A: Questionnaire used in the study

The questionnaire included items evaluating the presence and change of ocular symptoms (e.g., eye fatigue, dryness, photophobia, burning, foreign body sensation, tearing, itchiness, and blinking frequency) before and after using blue-filter spectacle lenses for at least 12 months.

Each symptom was rated on a four-point Likert-type scale (more, same, less, or no complaint).

Participants also provided information about daily screen time, number of digital devices used, adherence to the 20-20-20 rule, and use of artificial tears.

**Table 5:** Questionnaire used in the study

Section 1: Demographic Information
Email address:
Gender:
☐ Female
☐ Male
Age (in years):
Education level:
☐ Primary school
☐ Middle school
☐ High school
☐ Bachelor’s degree
☐ Master’s degree
☐ Doctorate
Employment status:
☐ Employed
☐ Student
☐ Unemployed
☐ Retired
Section 2: Digital Device Use
Daily digital devices used:
☐ Smartphone
☐ Computer
☐ TV
☐ Gaming console
☐ Tablet
Total daily screen time:
☐ 2–4 hours
☐ 4–6 hours
☐ 6–8 hours
☐ 8 hours or more
Have you heard of the 20-20-20 rule?
☐ Yes
☐ No
If yes, do you apply it?
☐ Yes
☐ No
☐ Sometimes
Do you wear prescription glasses or contact lenses?
☐ Yes
☐ No
Do you regularly use artificial tears?
☐ Yes
☐ No
Section 3: Blue-Violet Light Filter Lens Use
How long have you been using blue-violet light filtering glasses?
☐ 1–3 months
☐ 3–6 months
☐ 6–12 months
☐ More than 12 months
Do you regularly wear your blue-violet light-filtering glasses while using digital devices?
☐ Yes
☐ No
Section 4: Symptom Change Assessment
Eye fatigue:
☐ More
☐ Same
☐ Less
☐ No complaint
Dry eye sensation:
☐ More
☐ Same
☐ Less
☐ No complaint
Itching:
☐ More
☐ Same
☐ Less
☐ No complaint
Excessive blinking:
☐ More
☐ Same
☐ Less
☐ No complaint
Light sensitivity:
☐ More
☐ Same
☐ Less
☐ No complaint
Blurred vision:
☐ More
☐ Same
☐ Less
☐ No complaint
Burning sensation:
☐ More
☐ Same
☐ Less
☐ No complaint
Stinging/foreign-body sensation:
☐ More
☐ Same
☐ Less
☐ No complaint
Watery eyes/tearing:
☐ More
☐ Same
☐ Less
☐ No complaint
Headache:
☐ More
☐ Same
☐ Less
☐ No complaint
